# Development of a Brazilian Portuguese instrument to assess knowledge, attitudes, and practices of pregnant women with diabetes at a Brazilian center

**DOI:** 10.61622/rbgo/2026rbgo7

**Published:** 2026-04-17

**Authors:** Caroline Vitoria Alves Amorim, Laura Sousa Coelho de Sá, Ênio Luis Damaso, Rafaela Alkmin da Costa, Tatiana Assunção Zaccara, Patrícia Moretti Rehder, Belmiro Gonçalves Pereira, José Paulo de Siqueira Guida

**Affiliations:** 1 Universidade Estadual de Campinas Departamento de Tocoginecología Campinas SP Brazil Departamento de Tocoginecología, Universidade Estadual de Campinas, Campinas, SP, Brazil.; 2 Universidade de São Paulo Faculdade de Medicina de Bauru Departamento de Medicina Bauru SP Brazil Departamento de Medicina, Faculdade de Medicina de Bauru, Universidade de São Paulo, Bauru, SP, Brazil.; 3 Universidade de São Paulo Faculdade de Medicina Departamento de Obstetrícia e Ginecologia São Paulo São Paulo Brazil Disciplina de Obstetrícia, Departamento de Obstetrícia e Ginecologia, Faculdade de Medicina, Universidade de São Paulo, São Paulo, São Paulo, Brazil.

**Keywords:** Gestational diabetes, Knowledge, Attitudes, Practice, Psychometric

## Abstract

**Objective:**

To develop and test an instrument in Brazilian Portuguese to evaluate KAP regarding GDM.

**Methods:**

We developed an instrument with 22 items (10 items for practice, 4 for knowledge and 8 for attitude), which was tested in a population of pregnant women (24 – 28 weeks) with GDM. We performed an Exploratory Factor Analysis to evaluate the items included in the instrument, the covariance among the domains evaluated by the instrument, and the robustness of the instrument.

**Results:**

One hundred seventy-five participants were included in this study (mean gestational age: 25.4±7.1 weeks). The majority of them did not present adequate KAP regarding diabetes. The average knowledge score was 0.84±1.02 points; the attitude score was 32.46±2.94; and the practice score was 4.65±1.65. The model with the best robustness to evaluate KAP was composed of 3 items for knowledge, 6 for attitude, and 5 for practice.

**Conclusion:**

The levels of KAP regarding diabetes among Brazilian pregnant women are low. The moderate association between knowledge and practice underscores the need for targeted educational interventions within prenatal care. Implementing a specific Brazilian Portuguese instrument to evaluate KAP among this population can bring evidence regarding the dimension of the problem in the country.

## Introduction

In Brazil, hyperglycemia in pregnancy is one of the most common complications during gestation: it affects approximately 18% of pregnant women in the country, and it is estimated that one in six births occurs in women with gestational hyperglycemia. It is associated with factors such as sedentary lifestyle, poor eating habits, smoking, medications, drug use, and obesity.^(1,2)^

Diagnostic criteria for Gestational Diabetes (GDM) are established by the International Diabetes and Pregnancy Study Group (IADPSG) and endorsed by the Brazilian Federation of Obstetrics and Gynecology Societies (FEBRASGO), Brazilian Society of Diabetes (SBD) and Pan-American Health Organization (PAHO). The Brazilian Ministry of Health also adopts these criteria for diabetes screening during pregnancy.

According to the above mentioned criteria, GDM is diagnosed when a woman presents fasting glucose levels ≥ 92 mg/dL and ≤ 125 mg/dL, and/or 75g Oral Glucose Tolerance Test (75g OGTT) values ≥ 180 mg/dL at one hour and ≥ 153 mg/dL at two hours after glucose overload. Overt diabetes is diagnosed when fasting glucose is ≥ 126 mg/dL or OGTT (75g) values exceed 200 mg/dL in the second hour.^(3)^

Diabetes during pregnancy is a significant public health issue, as it is associated with preeclampsia, preterm birth, delivery complications such as obstructed labor, increased need for cesarean section and shoulder dystocia, and a higher risk of developing type 2 diabetes mellitus (T2DM) for both mother and child in the future. It is also linked to increased risk of neonatal complications, including malformations, fetal macrosomia, neonatal hypoglycemia, and jaundice.^(4,5)^

Treatment includes lifestyle modifications, blood glucose monitoring, and, in some cases, the use of insulin or other medications. GDM usually resolves after childbirth, but women with a history of GDM are at increased risk of developing T2DM after pregnancy and/or experiencing recurrence of GDM in future pregnancies.^(6)^

Therefore, pregnant women must have access to adequate information about the risks associated with diabetes in pregnancy and previous GDM. They must understand the preventive measures that can be taken to minimize these risks, along with regular medical follow-up and adopting a healthy lifestyle to prevent disease progression. This requires self-awareness and a positive attitude toward the diagnosis.^(7,8)^

This study aimed to understand the level of Knowledge, Attitude, and Practice (KAP) of pregnant women with diabetes, through the development and testing of an instrument in Brazilian Portuguese to assess the KAP.

## Methods

This cross-sectional study was conducted at the Women's Hospital of the University of Campinas (CAISM), Brazil, from March 2024 to February 2025. We followed these inclusion criteria: gestational age between 24 a 28 weeks and a diagnosis of diabetes mellitus according to the Brazilian Ministry of Health (fasting glucose ≥ 92 mg/dL, or 75g OGTT with any of the following values: fasting glucose ≥ 92 mg/dL, 1-hour ≥ 180 mg/dL, or 2-hour ≥ 153 mg/dL). These tests are part of routine prenatal care. Women under 18 years of age were excluded.

The estimated population prevalence of diabetes was 10.2%.^(2)^ The study center receives approximately 1,560 prenatal cases per year. Therefore, a sample size of 175 women was estimated. Participants were selected by convenience sampling among those attending the clinic during the data collection period until the required sample size was reached.

To assess diabetes-related practices, knowledge, and attitudes, the researchers interviewed participants using an instrument specifically developed for this study. The instrument was previously evaluated by four specialists not directly involved in this research.

The instrument was developed based on tools previously published in English.^(7,9,10)^ Two study authors, experienced in diabetes care and research and fluent in English, reviewed the three considered instruments, selected the most relevant items, and translated them into Brazilian Portuguese.

Subsequently, the initial version of the instrument was evaluated by four independent experts who had no prior knowledge of the study or the instrument. These experts were invited to assess the items regarding their translation from English to Portuguese and their contextual relevance to the Brazilian setting. Items approved by all experts were retained; the study authors discussed items approved by three experts; items rejected by two experts were excluded from the study. The instrument included 10 items for practices, 4 for knowledge, and 8 for attitudes. The questions are presented in tables 1, 2, and 3. The translation for Brazilian Portuguese is provided as a supplementary file. Higher scores indicate better practice, knowledge, or attitude toward diabetes.

- Practices: Participants received 1 point for each "Yes" or "Frequently" response, and 0 for each "No," "Occasionally," or "Never" response. Possible scores ranged from 0 to 10. Higher scores indicate more appropriate diabetes-related practices.
- Knowledge: Participants received 1 point for each correct answer regarding diagnostic and glycemic control criteria. The total possible score ranged from 0 to 4, with higher scores indicating greater knowledge.
- Attitudes: Participants rated their agreement with statements about diabetes on a 5-point Likert scale (Strongly Agree, Agree, Neutral, Disagree, Strongly Disagree). Each response was scored from 1 to 5, with Strongly Agree scoring 5 and Strongly Disagree scoring 1 (except for question A7, in which scoring was reversed). Total possible scores ranged from 8 to 40, with higher scores reflecting more positive attitudes toward diabetes.

**Table 1:** Knowledge regarding glucose goals among Brazilian pregnant women with diabetes

Question		Yes n(%)	No n(%)
K1	Criteria for good control of fasting blood glucose in a patient with GDM (correct answer: ≤ 95mg/dL)	32(18.29)	143(81.71)
K2	Criteria for good control of 1 h postprandial blood glucose in a patient with GDM (correct answer: ≤ 140mg/dL)	68(38.86)	107(61.14)
K3	Criteria for good control of 2 h postprandial blood glucose in a patient with GDM (correct answer: ≤ 120mg/dL)	34(19.43)	141(80.57)
K4	What is the maximum percentage of values that can be outside the target for adequate diabetes control? (correct answer: ≤ 30%).	13(7.47)	162(92.57)

**Table 2:** Attitude assessment on diabetes among Brazilian pregnant women

Question		Strongly agree n(%)	Agree n(%)	Neutral n(%)	Disagree n(%)	Strongly disagree n(%)
A1	Blood glucose monitoring throughout pregnancy is essential for patients with gestational diabetes mellitus (GDM)	94(53.71)	75(42.86)	3(1.71)	2(1.14)	1(0.57)
A2	The treatment of GDM includes nutritional therapy, movement therapy, insulin therapy, education on diabetes mellitus, and blood glucose monitoring.	78(44.57)	92(52.57)	5(2.86)	0	0
A3	All patients with GDM should receive a glucose tolerance test at 6–12 weeks after delivery.	25(14.29)	47(26.86)	93(53.14)	9(5.14)	1(0.57)
A4	An individualized management regimen is required for the treatment of GDM.	51(29.14)	118(67.43)	4(2.29)	2(1.14)	0
A5	Individualized guidance by medical staff can provide more reliable information than newspapers, television shows or other media and thus should be followed.	90(51.43)	82(46.86)	2(1.14)	1(0.57)	0
A6	The Nutritional Clinic plays an important role in the management of patients with GDM.	70(40.00)	99(56.57)	4(2.29)	2(1.14)	0
A7	Insulin therapy and drug therapy are difficult to accept by patients.	37(21.14)	69(39.43)	23(13.14)	38(21.71)	8(4.57)
A8	Good blood glucose control can reduce the risk to the mother and fetus.	87(49.71)	86(49.14)	2(1.14)	0	0

**Table 3:** Practice assessment on diabetes among Brazilian pregnant women

Question		Yes / Often n(%)	No / Never / Occasionally n(%)
P1	Do you monitor your blood glucose level regularly?	142(81.14)	33(18.86)
P2	Do you have a habit of recording your diet and weight?	29(16.57)	146(83.43)
P3	Do you stick with exercise?	16(9.14)	159(90.86)
P4	Do you follow the instructions of doctors or dieticians to eat a low-glucose, low-fat and low-oil diet on a daily basis?	76(43.43)	99(56.57)
P5	Do you follow the instructions of doctors or dieticians to control your daily total dietary intake?	27(15.43)	148(84.57)
P6	Do you bring candy with you for possible hypotension when doing exercise?	30(17.14)	145(82.86)
P7	Do you actively acquire knowledge about GDM, its management and other relevant information regularly from doctors, books, the internet or APP?	94(53.71)	81(46.29)
P8	Would you ask the community medical service for help with controlling your blood glucose level?	116(66.29)	59(33.71)
P9	Would you see a doctor immediately if your blood glucose level was not well controlled?	111(63.43)	64(36.57)
P10	Do you receive prenatal examinations regularly?	172(98.29)	3(1.71)

Sociodemographic variables (age, education, occupation, and obstetric history) and diabetes type (type 1, type 2, or GDM) were also collected.

Data were analyzed using R Studio. Categorical variables were described as frequencies and percentages, while continuous variables were presented as means and standard deviations. An Exploratory Factor Analysis (EFA) was conducted for the instrument, estimating the factor loadings of each item and the covariances among the three domains. Factor loadings greater than 0.5 were considered moderate to strong and were retained in the model. Model fit was also assessed using the following indices: Comparative Fit Index (CFI), Tucker-Lewis Index (TLI), Root Mean Square Error of Approximation (RMSEA), and Standardized Root Mean Square Residual (SRMR).

The CFI compares the fit of the proposed model to that of an independent (null) model in which all variables are assumed to be uncorrelated. CFI values range from 0 to 1, with values above 0.90 indicating good fit and above 0.95 indicating excellent fit. The TLI adjusts the CFI by accounting for model complexity. Values above 0.90 are generally interpreted as indicating a good model fit. TLI can occasionally yield values below 0 or slightly above 1. The RMSEA estimates the lack of fit in a model compared to a perfect model. Values below 0.08 suggest reasonable fit, while values below 0.05 indicate excellent fit. A 90% confidence interval is commonly reported along with the RMSEA value. The SRMR measures the average standardized difference between observed and predicted correlations. Values less than 0.08 are considered indicative of a good fit.

This study was approved by the Research Ethics Committee of the University of Campinas before data collection (approval number #6764058, 77919224.0.0000.5404). All participants received information about the study and were included only after signing the informed consent form.

## Results

A total of 175 participants were included, with a mean age of 31.3±6.4 years. Among them, 8 (4.57%) had type 1 diabetes, 26 (14.86%) had type 2 diabetes, and 141 (80.57%) had GDM. The mean gestational age at enrollment was 25.4±7.1 weeks. Regarding diabetes knowledge, 32 (17.8%) participants knew the correct fasting glucose threshold, 68 (39.1%) the 1-hour postprandial value, and 34 (19.5%) the 2-hour value. Thirteen participants (7.5%) knew the target values for adequate glycemic control. These results are presented in table 1.

As for attitudes, most women agreed with positive statements regarding diabetes care during pregnancy. Most recognized the importance of blood glucose monitoring and acknowledged that diabetes treatment involves diet, physical activity, health education, and insulin use. The majority also understood that reasonable metabolic control improves perinatal outcomes. However, many women did not recognize the importance of post-delivery diagnostic confirmation, and most reported that insulin treatment can be challenging. These results are shown in table 2.

Most women reported regular blood glucose monitoring (141, 81.03%), but only 29 (16.67%) reported keeping records of their diet and weight, and 16 (9.20%) reported engaging in physical activity. Most participants sought information about diabetes (93, 53.45%) and participated in activities at their local health centers (115, 66.09%) to improve disease management. There was high adherence to prenatal visits (171, 98.28%). These results are presented in table 3.

The average knowledge score was 0.84±1.02 points; the attitude score was 32.46±2.94; and the practice score was 4.65±1.65. We tested three models of EFA. The first was a compound of all the instrument's items. The results of this EFA demonstrated a reasonable fit (CFI = 0.811; TLI = 0.788; RMSEA = 0.051; SRMR = 0.074). Regarding the Knowledge factor, one item presented a weak loading, while the remaining items showed acceptable factor loadings. For the Attitudes factor, two items exhibited weak loadings. In the Practices factor, six items had weak loadings. The covariance analysis among the latent factors revealed a moderate correlation between Knowledge and Practice (0.243), but no significant correlations between Knowledge and Attitude (0.152), nor between Attitude and Practice (0.131). This data is presented in table 4. In the second model, we included three items of Knowledge, six items of Attitude, and seven items of Practice. We observed that CFI (0.872) and TLI (0.848) were below 0.90, suggesting that the model is not well-adjusted. The RMSEA (0.058) and SRMR (0.070) were acceptable. The items included for Knowledge had a moderate to high factor loading, and the same occurred for most of the items included for Attitude. For Practice, two items had a low factor loading. No significant covariance between the categories evaluated was observed. The results are presented in table 4.

**Table 4:** Exploratory Factorial Analysis of an instrument in Brazilian Portuguese developed to evaluate Knowledge, Attitude and Practice (KPA) among pregnant women with diabetes

Item		Factor loading	Factor loading	Factor loading
Model		Model 1	Model 2	Model 3
	K1	0.450	0.417	0.418
	K2	0.738	0.811	0.802
	K3	0.469	0.435	0.441
	K4	0.230	Excluded	Excluded
Attitude				
	A1	0.649	0.648	0.649
	A2	0.779	0.777	0.777
	A3	0.204	Excluded	Excluded
	A4	0.500	0.496	0.497
	A5	0.660	0.662	0.660
	A6	0.561	0.555	0.555
	A7	0.019	Excluded	Excluded
	A8	0.676	0.687	0.686
Practice				
	P1	0.236	0.242	0.285
	P2	0.495	0.521	0.539
	P3	0.143	Excluded	Excluded
	P4	0.567	0.558	0.542
	P5	0.572	0.562	0.559
	P6	0.025	Excluded	Excluded
	P7	0.440	0.430	0.400
	P8	0.106	Excluded	Excluded
	P9	0.440	0.310	Excluded
	P10	0.186	0.183	Excluded
Covariances				
	Knowledge and Attitude	0.152	0.134	0.137
	Knowledge and Practice	0.243	0.238	0.277
	Attitude and Practice	0.131	0.117	0.152
	CFI	0.811	0.872	0.922
	TLI	0.788	0.848	0.904
	RMSEA	0.051	0.058	0.050
	SRMR	0.074	0.070	0.065

We excluded two items for Practice in the third model and kept the other items included in model 2. We observed that CFI (0.922) and TLI (0.904) increased, suggesting this is a more robust instrument version. The RMSEA (0.055) and SRMR (0.065) were acceptable.

## Discussion

This study provides valuable insights into the KAP of pregnant women with diabetes in a Brazilian tertiary care setting. The majority of participants had GDM, which reflects the expected epidemiological profile in Brazil, where GDM is a common condition during pregnancy.^(1)^ Our study also proposed an instrument in Brazilian Portuguese to evaluate the KAP in our population.

The overall knowledge about diabetes and glycemic targets among participants was low. Less than 20% of women correctly identified fasting or 2-hour postprandial glucose thresholds, and only 7.5% knew the recommended values for adequate glycemic control. This knowledge gap is concerning, given the established association between hyperglycemia in pregnancy and adverse maternal and neonatal outcomes. Previous studies have also reported insufficient diabetes knowledge among pregnant women, especially in low- and middle-income countries, where education gaps and healthcare access disparities are common.^(11,12)^ The findings underscore the need for more effective educational strategies, integrated into routine antenatal care, to ensure that patients understand the importance of glycemic targets and their role in self-management.

Despite high adherence to certain clinical recommendations, such as regular attendance at prenatal visits and blood glucose monitoring, the study identified low implementation of key behavioral practices, such as physical activity and dietary tracking. This discrepancy suggests that presence in healthcare services alone does not ensure the adoption of necessary lifestyle changes for adequate diabetes management during pregnancy.^(13,14)^

Factors such as time constraints, multiple domestic responsibilities, limited access to personalized nutritional counseling, and sociocultural barriers may hinder the ability to translate positive attitudes into practical actions. These findings underscore the need for structured, ongoing, patient-centered interventions, supported by interprofessional collaboration and educational tools to promote sustainable behavioral changes.^(13,14)^

Despite limited knowledge, participants generally expressed positive attitudes toward diabetes care. Most recognized the importance of monitoring blood glucose and accepted that effective management involves lifestyle changes and medical treatment. These positive attitudes may represent a critical foundation for behavioral change, even when lacking knowledge. However, the perception that insulin treatment is complex and the lack of awareness about the importance of post-delivery diagnostic follow-up highlight areas where patient counseling could be improved.^(15,16)^

In terms of practices, while the majority reported regular blood glucose monitoring, fewer engaged in recommended behaviors such as physical activity and dietary tracking. This disconnect between positive attitudes and actual practices may reflect structural barriers such as time constraints, limited access to nutritional counseling or exercise programs, or lack of support. These findings align with previous reports suggesting that while motivation may exist, effective implementation of diabetes self-care behaviors during pregnancy is often hindered by contextual factors.^(13,17)^

The CFA indicated a reasonable model fit, supporting the validity of the proposed KAP framework. However, several items — particularly within the Practices domain — showed weak factor loadings. This suggests the need to refine the instrument to better capture the complexity and variability of diabetes-related behaviors during pregnancy. The moderate correlation between Knowledge and Practice reinforces the idea that improving knowledge may positively influence behavior. However, attitudes did not significantly correlate with either domain, suggesting that they operate independently or are influenced by external factors not captured by the instrument.

This instrument can be used for both research and clinical purposes. In clinical practice, it is possible that, in the future, this tool may be applied after the diagnosis and initial consultation of pregnant women with diabetes. These women would have their KAP levels systematically assessed, and those with low KAP scores could undergo targeted interventions aimed at improving their knowledge.

The future use of this instrument in research will also allow for an understanding of whether high KAP scores are associated with better perinatal outcomes. This will be the focus of future studies that will evaluate the prospective application of the tool at different stages of prenatal care.

This study has some limitations. It was conducted in a single tertiary care center, which may limit generalizability to other settings. The use of a convenience sample and self-reported measures may also introduce bias. The findings offer important implications for clinical practice and public health interventions. Structured diabetes education, delivered through culturally appropriate and patient-centered approaches, may improve knowledge and empower women to adopt healthier practices during pregnancy.

## Conclusion

The findings of this study highlight significant gaps in knowledge and self-care practices among pregnant women with diabetes, despite generally positive attitudes toward disease management. While most participants understood the importance of glucose control, few were aware of specific glycemic targets or consistently engaged in recommended behaviors such as physical activity and dietary monitoring. The moderate association between knowledge and practice underscores the need for targeted educational interventions within prenatal care. Improving health literacy and supporting behavior change through structured, context-sensitive strategies may enhance maternal and neonatal outcomes in this vulnerable population.

## Data Availability

The research data are described in the article presented.
