# Supplementary material for: Development of a Brazilian Portuguese instrument to assess knowledge, attitudes, and practices of pregnant women with diabetes at a Brazilian center
Source: Rev Bras Ginecol Obstet. 2026 Apr 17;48:e-rbgo7. doi: 10.61622/rbgo/2026rbgo7 (PMC13078509; doi:10.61622/rbgo/2026rbgo7)
Supplement: SUPPLEMENTARY MATERIAL [file 1806-9339-rbgo-48-e-rbgo7-suppl1.pdf]

Supplementary material. English and Portuguese versions of the Instrument

Item	English	Item	Português
Section	Basic Information	Seção	Informações Básicas
1	Your Age	1	Sua idade
2	Your Education	2	Sua escolaridade
	Middle School or below		Ensino fundamental completo ou incompleto
	High School / Technical School		Ensino médio completo ou incompleto
	Junior college / college		Ensino superior completo ou incompleto
	Master's degree or higher		Pós-Graduação
3	Your occupational category	3	Sua ocupação principal
4	Your Pre-pregnancy Weight (kg)	4	Seu peso antes da gravidez (kg)
	Height cm		Altura (cm)
5	Gravidity	5	Número de gestações (contando a atual)
6	Parity	6	Número de partos vaginais
		7	Número de cesáreas prévias
		8	Número de abortos
7	Method of pregnancy:	9	Forma da gestação
	Natural conception		Espontânea
	Assisted reproduction		Fertilização
8	Do you have Polycystic Ovary Syndrome (PCOS)?	10	Você tem síndrome dos ovários policísticos?
	Yes		Sim
	No		Não
9	Do you have a Family History of Diabetes?	11	Você tem antecedente familiar (mãe, pai, irmãs/irmãos ou filhas/filhos) com diabetes?
	Yes		Sim
	No		Não
10	Have you had a history of Macrosomia before (large infant at birth)?	12	Você tem antecedente de bebê com macrossomia (ou bebê que nasceu muito grande, acima de 4kg)?
	Yes		Sim
	No		Não
		13	Você tem antecedente de hipertensão crônica / pressão alta antes da gestação?
			Sim
			Não
		14	Você está fazendo uso de AAS (Aspirina)?
			Sim
			Não
		15	Você está fazendo uso de carbonato de cálcio?
			Sim
			Não
		16	Em relação ao diabetes, seu diagnóstico atual é de:
			Diabetes Gestacional
			Diabetes tipo 1
			Diabetes tipo 2
11	Current Gestational Week	17	Idade gestacional atual (em semanas completas)
	**Knowledge Assessment**		**Avaliação do Conhecimento**
1	Criteria for good control of fasting blood glucose in a patient with GDM	1	Qual o valor máximo de dextro (ponta de dedo / glicemia capilar) para o controle adequado no jejum?<br><br>(Resposta correta = 95)
2	Criteria for good control of 1 h postprandial blood glucose in a patient with GDM	2	Qual o valor máximo de dextro (ponta de dedo / glicemia capilar) para o controle adequado após 1 hora da refeição (café da manhã, almoço ou jantar)?<br><br>(Resposta correta = 140)
3	Criteria for good control of 2 h postprandial blood glucose in a patient with GDM	3	Qual o valor máximo de dextro (ponta de dedo / glicemia capilar) para o controle adequado após 2 horas da refeição (café da manhã, almoço ou jantar)?<br><br>(Resposta correta = 120)
		4	Qual o percentual máximo de valores que podem estar fora da meta para um adequado controle do diabetes?<br><br>(Resposta correta = 30%)
	**Attitude Assessment**		**Avaliação das Atitudes**
1	Blood glucose monitoring throughout pregnancy is essential for patients with gestational diabetes mellitus (GDM)	1	A monitorização dos níveis glicêmicos durante a gravidez é essencial para pacientes com diabetes gestacional.
	Strongly agree		Concordo fortemente

Continue...

Continuation.

Item	English	Item	Português
	Agree		Concordo
	Neutral		Neutro
	Disagree		Discordo
	Strongly disagree		Discordo fortemente
2	The treatment of GDM includes nutritional therapy, movement therapy, insulin therapy, education on diabetes mellitus, and blood glucose monitoring.	2	O tratamento do Diabetes Gestacional inclui dieta adequada, atividade física, uso de insulina, controle glicêmico e educação sobre a doença.
	Strongly agree		Concordo fortemente
	Agree		Concordo
	Neutral		Neutro
	Disagree		Discordo
	Strongly disagree		Discordo fortemente
3	All patients with GDM should receive a glucose tolerance test at 6–12 weeks after delivery.	3	Todas pacientes com Diabetes Gestacional devem realizar teste de tolerância oral a glicose de 6 a 12 semanas após o parto.
	Strongly agree		Concordo fortemente
	Agree		Concordo
	Neutral		Neutro
	Disagree		Discordo
	Strongly disagree		Discordo fortemente
4	An individualized management regimen is required for the treatment of GDM.	4	Um tratamento individualizado é necessário para o tratamento do Diabetes Gestacional.
	Strongly agree		Concordo fortemente
	Agree		Concordo
	Neutral		Neutro
	Disagree		Discordo
	Strongly disagree		Discordo fortemente
5	Individualized guidance by medical staff can provide more reliable information than newspapers, television shows or other media and thus should be followed.	5	O tratamento individualizado por equipe de saúde pode proporcionar informações mais confiáveis que jornais, revistas e mídias sociais.
	Strongly agree		Concordo fortemente
	Agree		Concordo
	Neutral		Neutro
	Disagree		Discordo
	Strongly disagree		Discordo fortemente
6	The Nutritional Clinic plays an important role in the management of patients with GDM.	6	O nutricionista tem um papel importante no cuidado das mulheres com diabetes gestacional.
	Strongly agree		Concordo fortemente
	Agree		Concordo
	Neutral		Neutro
	Disagree		Discordo
	Strongly disagree		Discordo fortemente
7	Insulin therapy and drug therapy are difficult to accept by patients.	7	O tratamento com insulina e outros medicamentos é difícil de ser aceito pelas pacientes.
	Strongly agree		Concordo fortemente
	Agree		Concordo
	Neutral		Neutro
	Disagree		Discordo
	Strongly disagree		Discordo fortemente
8	Good blood glucose control can reduce the risk to the mother and fetus.	8	Um bom controle glicêmico pode reduzir os riscos para a mãe e o bebê.
	Strongly agree		Concordo fortemente
	Agree		Concordo
	Neutral		Neutro
	Disagree		Discordo
	Strongly disagree		Discordo fortemente
	**Practice Assessment**		**Avaliação das Práticas**
1	Do you monitor your blood glucose level regularly?	1	Você faz o monitoramento dos níveis de glicose regularmente?
	Never		Nunca
	Occasionally		Ocasionalmente
	Frequently		Frequentemente
2	Do you have a habit of recording your diet and weight?	2	Você tem o hábito de registrar sua dieta e seu peso?
	Never		Nunca
	Occasionally		Ocasionalmente
	Frequently		Frequentemente
3	Do you stick with exercise?	3	Você faz exercícios?
	Never		Nunca

Continue...

Continuation.

Item	English	Item	Português
	Occasionally		Ocasionalmente
	Frequently		Frequentemente
4	Do you follow the instructions of doctors or dieticians to eat a low-glucose, low-fat and low-oil diet on a daily basis?	4	Você segue as orientações de médicos e nutricionistas quanto à composição da dieta quanto à carboidratos, proteínas e gorduras?
	Never		Nunca
	Occasionally		Ocasionalmente
	Frequently		Frequentemente
5	Do you follow the instructions of doctors or dieticians to control your daily total dietary intake?	5	Você segue as orientações de médicos e nutricionistas quanto à quantidade total de calorias?
	Never		Nunca
	Occasionally		Ocasionalmente
	Frequently		Frequentemente
6	Do you bring candy with you for possible hypotension when doing exercise?	6	Você leva um doce com você quando vai praticar atividade física?
	Yes		Sim
	No		Não
7	Do you actively acquire knowledge about GDM, its management and other relevant information regularly from doctors, books, the internet or APP?	7	Você busca informações sobre o Diabetes gestacional com seu médico, em livros, internet, mídias sociais, etc?
	Never		Nunca
	Occasionally		Ocasionalmente
	Frequently		Frequentemente
8	Would you ask the community medical service for help with controlling your blood glucose level?	8	Você busca ajuda na unidade básica de saúde para um melhor controle da sua glicemia?
	Yes		Sim
	No		Não
9	Would you see a doctor immediately if your blood glucose level was not well controlled?	9	Você procura um médico imediatamente se observa um descontrole nos seus níveis glicêmicos?
	Yes		Sim
	No		Não
10	Do you receive prenatal examinations regularly?	10	Você comparece regularmente às consultas de pré-natal?
	Yes		Sim
	No		Não
